# EGFR exon 20 insertion mutations and response to osimertinib in non-small-cell lung cancer

**DOI:** 10.1186/s12885-019-5820-0

**Published:** 2019-06-17

**Authors:** Wenfeng Fang, Yihua Huang, Shaodong Hong, Zhonghan Zhang, Minghui Wang, Jiadi Gan, Wenjing Wang, Honglin Guo, Kai Wang, Li Zhang

**Affiliations:** 1 Department of Medical Oncology, State Key Laboratory of Oncology in South China, Collaborative Innovation Center for Cancer Medicine, Sun Yat-Sen University Cancer Center, Guangzhou, 510060 People’s Republic of China; 2 Sun Yat-Sen Memorial Hospital, Sun Yat-Sen University, Guangzhou, 510220 People’s Republic of China; 3 OrigiMed, Inc, NO.115 XinJunhuan Road, Shanghai, 201114 People’s Republic of China

**Keywords:** NSCLC, EGFRex20ins, NGS, Osimertinib

## Abstract

**Background:**

Epidermal growth factor receptor exon 20 insertion (EGFRex20ins) mutations represent approximately 4–12% of EGFR mutations and are generally refractory to the 1st and 2nd generation EGFR tyrosine kinase inhibitors (TKIs). Development of effective therapies for patients with EGFRex20ins mutant non-small-cell lung carcinoma (NSCLC) represents a great unmet need. Preclinical models have shown that osimertinib is active in NSCLC harboring EGFRex20ins, while the antitumor activity of osimertinib remains to be evaluated in patients with EGFRex20ins mutations.

**Methods:**

Tumor genotyping was performed in 2316 Chinese NSCLC cases with targeted next generation sequencing (NGS) covering the whole exons of EGFR gene. The frequency and genetic characteristics of EGFRexon20ins mutations were analyzed. Furthermore, six patients with specific EGFRexon20ins mutations and receiving osimertinib 80 mg once daily were retrospectively included to assess the antitumor activity and safety of monotherapy osimertinib.

**Results:**

EGFRex20ins mutations were identified in 4.8% (53/1095) of EGFR mutant NSCLC and 2.3% (53/2316) of all NSCLC cases. The most frequently identified EGFRexon20ins is A767_V769dup (17/53,32.1%). We found that the genetic characteristics of EGFRex20ins mutations in Chinese patients with NSCLC were comparable to those reported in Caucasian patients. Four patients with osimertinib therapy achieved partial response and the rest stable disease. Median progression free survival (PFS) was 6.2 months (95% confidence interval 5.0–12.9 months; range 4.9–14.6 months). The most common adverse events (AEs) were diarrhea (2/6), pruritis (2/6), stomatitis (1/6) and nausea (1/6). No grade 3 or more AEs were documented.

**Conclusions:**

This study revealed that the genetic characteristics of EGFRex20ins mutations in Chinese patients with NSCLC were comparable to those reported in Caucasian patients. Furthermore, our study firstly demonstrated promising antitumor activity of osimertinib in certain EGFRex20ins mutant advanced NSCLC patients, indicating that osimertinib treatment for EGFRex20ins positive patients deserves further study.

**Electronic supplementary material:**

The online version of this article (10.1186/s12885-019-5820-0) contains supplementary material, which is available to authorized users.

## Background

During the past decades, the identification of specific genomic aberrations and their corresponding targeted therapies have significantly improved the outcome and quality of life for patients with non-small-cell lung cancer (NSCLC). Epidermal growth factor receptor (EGFR) mutation is the first identified targetable driver mutation that was reported in about 17 and 50% of lung adenocarcinoma in Caucasians and Asians, respectively [1–3]. The most common cluster of mutations in EGFR gene include inframe deletions around the LeuArgGluAla motif (residues 746–750) of exon 19, and the Leu858Arg (L858R) point mutation in exon 21, each accounting for about 45% of all EGFR mutations. These mutations are termed classic EGFR mutations and are more common in tumors in women, Asians, never smokers, and those with adenocarcinoma [4–6]. The frequency and distribution of EGFR mutations in patients with different ethnic backgrounds also differ [7, 8].

Patients with classic EGFR mutations generally have profound radiographic and clinical response to monotherapy EGFR tyrosine kinase inhibitors (TKIs) [9–12]. However, some unclassical EGFR mutations are associated with poor responses with reversible EGFR TKIs [13, 14]. Among these are most EGFR exon 20 insertion (EGFRex20ins) mutations reported as far. Exon 20 of EGFR encompasses nucleotides that translate into amino acid at position 762 to 823. It contains a C-helix (residues 762–766) and the loop following C-helix (residues 767–774), where the insertions could induce ligand-independent EGFR pathway activation and give rise to tumorigenesis [15]. The true frequency of EGFRex20ins mutations within the EGFR mutant lung cancer is inconsistent, contributing to roughly 4–12% of all EGFR mutations identified [16–18]. In most reports, EGFRex20ins mutations are more common in tumors among never-smokers [16, 18]. However, the genetic and clinical characteristics of NSCLCs harboring EGFRex20ins mutations in Asian populations remain unknown due to the lack of large comprehensive genomic studies.

Preclinical and clinical studies have shown that most EGFRex20ins (except for few subtypes such as EGFR A763_Y764insFQEA) mutant tumors confer resistance to the 1st and 2nd generation EGFR TKIs because the insertions produce steric hindrance and activate EGFR without saliently decreasing affinity for ATP or enhancing affinity for EGFR TKIs [15, 19–23]. Several clinical studies specifically involving tumors with EGFRex20ins mutations are ongoing, with some of them showing preliminary promising activity [24, 25]. However, there are still no established molecular targeted drugs for NSCLC patients with EGFRx20ins mutations. Development of more effective therapeutics for these specific patients represents a great unmet need.

Osimertinib is an oral, potent, irreversible EGFR TKI selective for sensitizing EGFR and EGFR T790 M resistance mutations. Preclinical studies have reported that osimertinib was active in EGFRex20ins mutant cell lines and tumor xenografts with a wide therapeutic window [26–29]. However, whether the preclinical activity of osimertinib could translate into clinical effect remains unclear.

Herein, we explored the characteristics of EGFRex20ins, as well as the patterns of co-mutations (mutually exclusive or inclusive) in Chinese NSCLC patients. In addition, we assessed the safety and antitumor activity of osimertinib in six advanced NSCLC patients with various EGFRex20ins mutations.

## Methods

### Patients

The study included a cohort of patients who were referred to OrigiMed (Shanghai, China) for targeted next generation sequencing (NGS) test in China between August 2016 and July 2018. Patient samples and clinical information including gender, age and histologic subtypes were retrieved at the time of referral. Six patients with EGFRex20ins mutant stage IV NSCLC who were treated with osimertinib 80 mg once daily were included to evaluate the antitumor activity of osimertinib. Data were retrospectively collected from digital medical records. The study was approved by the Institutional Review Board of SYSUCC and written informed consent was obtained for each patient prior to sample collection. Written consents were obtained from parents if patients were under 16 years old.

### EGFR ex20ins and co-mutations analysis

DNA from Formalin-fixed, paraffin-embedded tumor tissue and matched blood samples was extracted. Comprehensive genomic profiling was performed by NGS with a 37 or 450 cancer related gene panel covering the whole exons of EGFR gene at a mean coverage depth of >800X (1547 cases with 37 panel, and 769 cases with 450 panel). The genomic alterations including single base substitution, insertions/deletions, copy number variations, as well as gene rearrangement and fusions were assessed. As for six patients treated with osimertinib, genetic status was also determined through NGS prior to osimertinib.

### Response evaluation

All six patients received oral osimertinib 80 mg once daily. Radiological follow-up was performed at the first months then once every 2 months with computed tomography (CT) of the thorax and upper abdomen. Regular cerebral magnetic resonance imaging (MRI) with CT was carried out once any patient was confirmed brain metastasis. Response was assessed according to Response Criteria in Solid Tumors (RECIST) 1.1 [30]. Progression-free survival (PFS) was defined as the interval from the date of initiation of osimertinib therapy to the date of disease progression or death from any cause, whichever occurred first.

### Statistical analyses

The statistical analyses were performed using the SPSS 20.0 (Chicago, IL, USA). The difference in the frequency of each group was analyzed by the Chi-square test or Fisher’s exact test. The median age between groups was compared using nonparametric test. A two-sided *p* value < 0.05 was considered statistically significant.

## Results

### Frequency and genetic characteristics of EGFRex20ins mutations

**Fig. 1:**
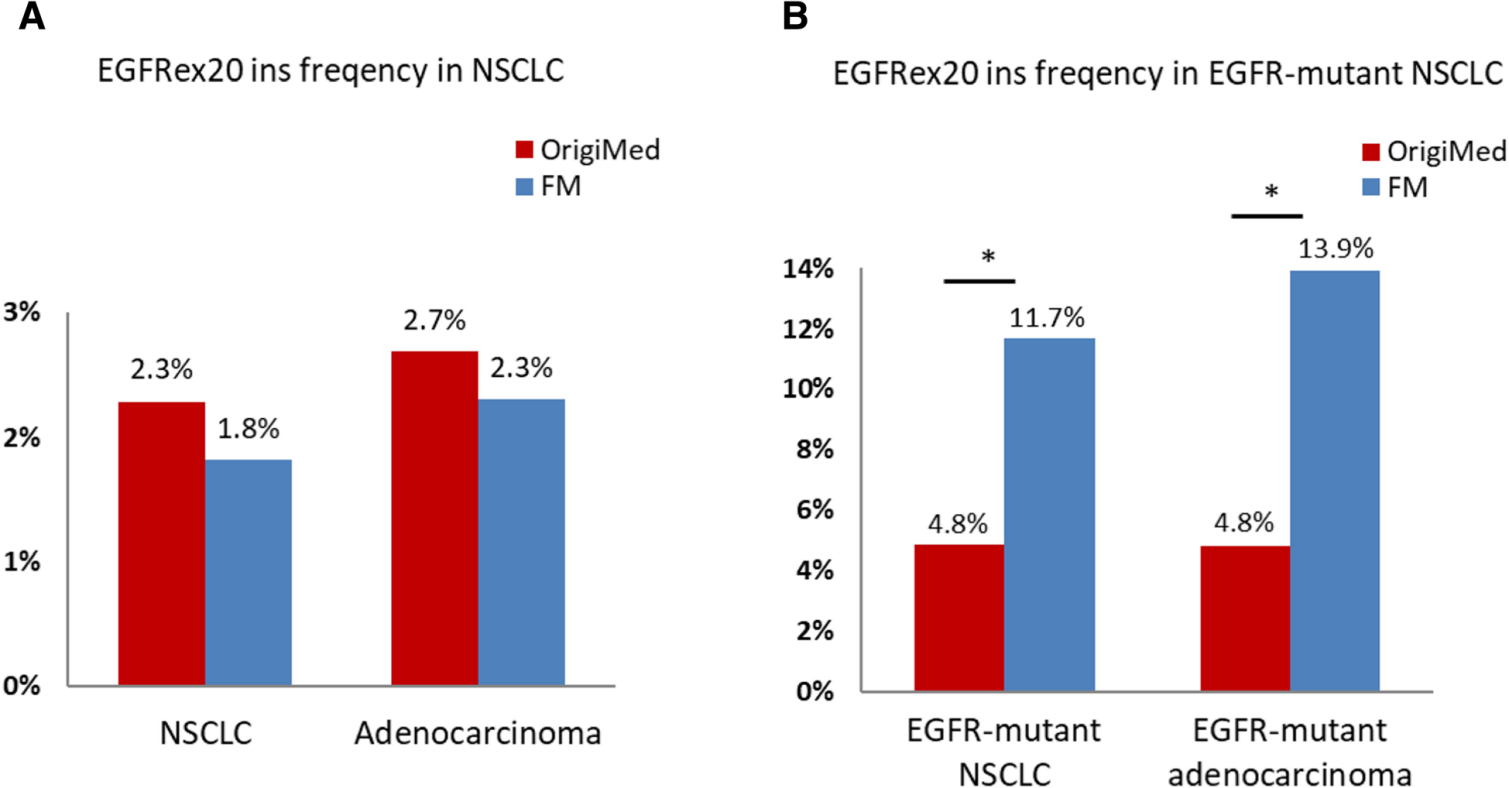
Frequency of EGFRex20ins mutations. **a**. Comparison of EGFRex20ins frequency in total NSCLC patients (OrigiMed 2.3% vs. FM 1.8%, *p* = 0.12) and adenocarcinoma patients (OrigiMed 2.7% vs. FM 2.3%, *p* = 0.32). **b**. Comparison of EGFRex20ins frequency in EGFR-mutant NSCLC patients (OrigiMed 4.8% vs. FM 11.7%, *p* < 0.001) and EGFR-mutant adenocarcinoma patients (OrigiMed 4.8% vs. FM 13.9%, *p* < 0.001). EGFRex20ins, epidermal growth factor receptor exon 20 insertions; NSCLC, non-small cell lung cancer; FM: Foundation Medicine. * *p* < 0.001

Among the 2316 unselective NSCLC tumors, EGFR mutations were identified in 1095 cases (47.3%). EGFRex20ins mutations were detected in 53 cases, contributing 2.3% of all NSCLC cases and 4.8% of EGFR-mutant tumors. Compared with Foundation Medicine (FM) data representing the largest EGFRex20ins cohort, we found that although EGFR mutations were much more common in our Chinese NSCLC patients than that of Western population (47.3% vs 15.5% in FM), EGFRex20ins mutation represented a much smaller group in EGFR mutant NSCLC (4.8% vs 11.7% in FM, *p* < 0.001) [17]. Of note, our result was comparable with FM cohort in frequency of EGFRex20ins in total NSCLC (2.3% vs. 1.8% in FM, *p* = 0.12, Fig. 1). The smaller proportion of EGFRex20ins in Chinese populations is due to the larger scale of EGFR mutations than western groups.

**Fig. 2:**
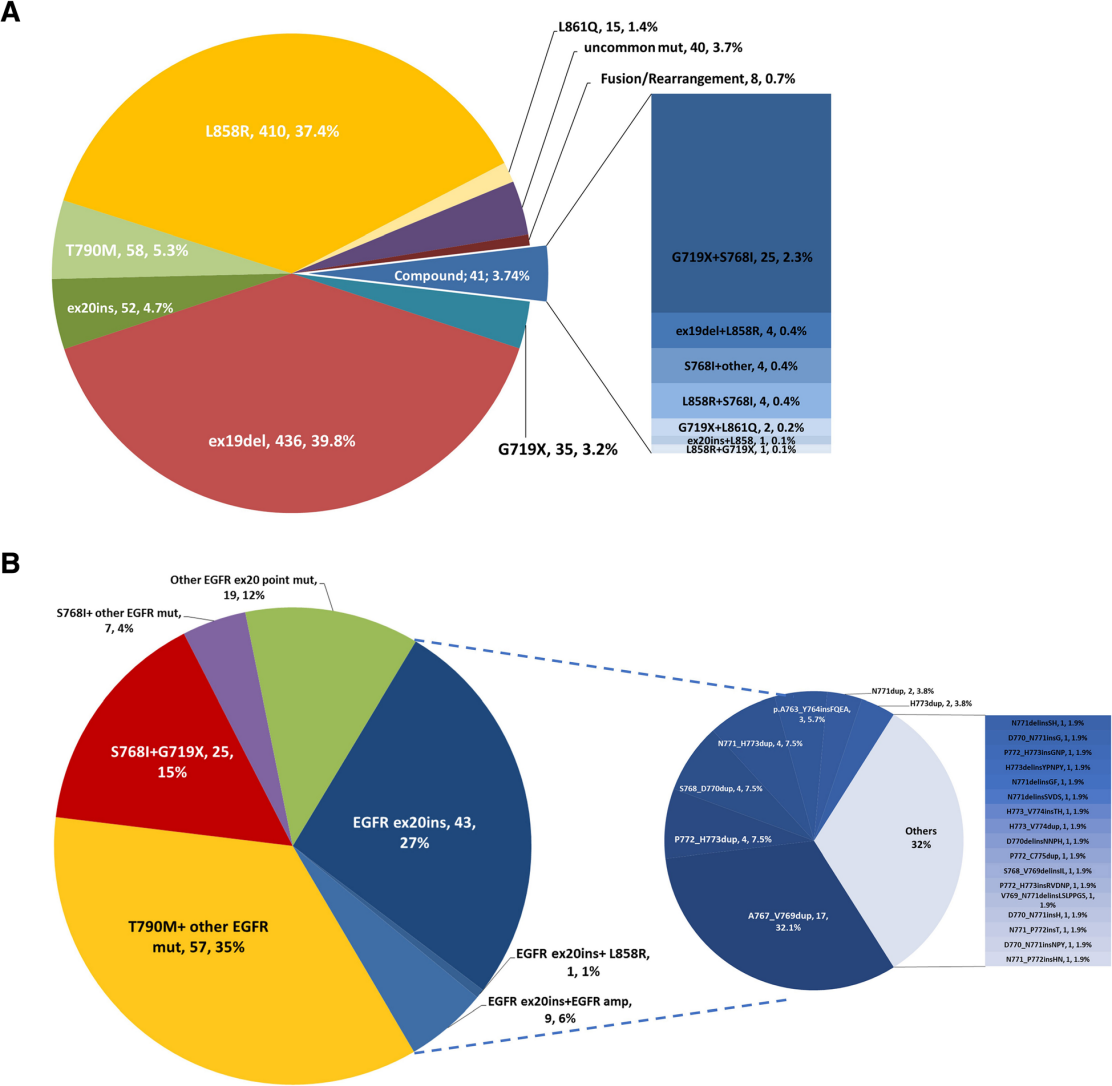
Distribution of EGFR mutations and EGFR exon 20 mutation types and EGFRex20ins in this study. **a**. Distribution of EGFR mutations. **b**. Distribution of EGFR exon 20 mutation types and EGFRex20ins mutations

The demographic and clinical characteristics of these patients are summarized in Tables 1 and 2. Of the patients with EGFR mutations, EGFRex20ins ranked the fourth most common type, following EGFR exon 19 deletions (436/1095, 39.8%), L858R (410/1095, 37.4%) and T790 M mutations (58/1095, 5.3%) (Fig. 2a). The majority of EGFRex20ins mutations were identified in lung adenocarcinoma (92.5%, 49/53). EGFRex20ins were also detected in two adenosquamous cases and two NSCLC not otherwise specified (NOS). Median age of patients with exon 20 insertion is 57 (31–85) years.

**Table 1:** Histologic and clinical characteristics of non-small cell lung cancer patients tested in this study

	Adenocarcinoma	Squamous	Others ^a^	Total
Total cases	1820	290	206	2316
Median age	60(26–92)	62(30–88)	60(12–83)	61(12–92)
Sex, M/F	917/903	247/43	144/62	1308/1008
EGFR mutant cases	1021	11	63	1095
Frequency in total	56.1%	3.8%	30.6%	47.3%
Median age	60(26–86)	64(38–89)	57(27–74)	60(26–89)
Sex, M/F	397/624	5/6	28/35	430/665
EGFRex20ins cases	49	0	4	53
Frequency in EGFR mutant	4.8%	0	6.3%	4.8%
Frequency in total	2.7%	0	1.9%	2.3%
Median age	57(31–85)	–	56.5(49–70)	57(31–85)
Sex, M/F	26/23	–	2/2	28/25

^a^ Other pathological type in NSCLC, including adenosquamous lung cancer, NSCLC not otherwise specified, large cell lung cancer, neuroendocrine carcinoma and sarcomatoid carcinoma

*M/F* male/female; EGFRex20ins, epidermal growth factor receptor exon 20 insertions, *NSCLC* non-small cell lung cancer

**Table 2:** Clinical comparison of EGFR ex20ins NSCLC with EGFR

	EGFR 20ins	EGFR WT	EGFR 19del	EGFR L858R	EGFR T790 M
Median age	57	61	59.5	62	59
*p* value vs. EGFR ex20ins		0.399	0.897	0.206	0.792
Sex, M/F(%M)	28/25(53%)	878/343(72%)	177/259(41%)	130/280(32%)	21/37(36%)
*p* value vs. EGFR ex20ins		0.003	0.088	0.002	0.078

WT and EGFR mutant (19del/L858R/ T790 M) NSCLC

*WT*wild type

In total, 20 different variants of exon EGFRex20ins were identified in 53 NSCLC patients. The most frequent variant is A767_V769dup (32.1%, 17/53), followed by P772_H773dup (4/53, 7.5%), S768_D770dup (4/53, 7.5%), N771_H773dup (4/53, 7.5%), A763_Y764insFQEA (3/53, 5.7%). Unique EGFRex20ins mutations detected by NGS were summarized in Fig. 2b.

EGFRex20ins tended to be exclusive with NSCLC driver genes such as *EGFR* mutation ERBB2, ALK, BRAF and RET mutations. The most common co-mutations were TP53 (49.1%). Co-mutation pattern compared with EGFR ex19dels, L858R, T790 M and other EGFR uncommon mutations were summarized in Additional file 1: Figure S1 and Additional file 2: Figure S2.

### Antitumor activity of monotherapy Osimertinib for patients with EGFRex20ins mutations

From August 28th, 2017 to April 30th, 2018, six patients with stage IV lung adenocarcinoma bearing EGFRex20ins started osimertinib treatment. Median follow-up time was 6.2 months. Previous treatment, detailed mutation characteristics and the outcome of osimertinib are shown in Table 3. All the patients had stage IV lung adenocarcinoma and predominantly females (5/6). Before osimertinib treatment, four patients were observed metastasis in lungs and pleura. Patient 5 was diagnosed with brain metastasis and patient 6 with bone metastasis. The median age is 64 years old. Two patients received osimertinib as first line therapy and two patients had previous treatment with other EGFR TKIs. Per RECISIT 1.1, four (67.7%) patients achieved partial response (PR) and the remaining two patients (33.3%) obtained stable disease (SD). Median progression-free survival (PFS) was 6.2 months (95% confidence interval 5.0–12.9 months; range 4.9–14.6 months). Treatment-related adverse events (AEs) included diarrhea (2/6), pruritus (2/6), stomatitis (1/6) and nausea (1/6). No grade 3 or more AEs were documented. At data cut-off (December 1st, 2018), Two patients had sustaining disease control and remained on osimertinib treatment, while the other four patients had progressive disease (PD) ultimately.

**Table 3:** Mutation characteristics and outcome of osimertinib treatment

Patient	Age	Sex	Previous Systematic therapy	Previous EGFR TKI Treatment	Mutations ^a^ Before osimertinib (MAF)	Best Response	PFS
1	63Y	F	None	None	EGFR p. A767_V769dup (3.48%)	PR	6.0 m
2	59Y	F	Yes	None	EGFR p. S768_D770dup (1.75%)	PR Treatment ongoing	14.6 m
3	69Y	M	None	None	EGFR p. N771_P772insL (47%)	SD	4.9 m
4	70Y	F	Yes	afatinib	EGFR p. S768_D770dup (3%)	SD Treatment ongoing	11.2 m
5	63Y	F	Yes	None	EGFR p. D770_N771insG (24%)	PR	6.4 m
6	65Y	F	None	gefitinib	EGFR p. A763_Y764insFQEA (0.2%) T790 M (0.3%)	PR	5.1 m

^a^ Mutations predicting EGFR TKI treatment

*TKI* tyrosine kinase inhibitor, *MAF* mutation allele fraction, *PFS* progression free survival, *PR* partial response, *SD* stable disease

**Fig. 3:**
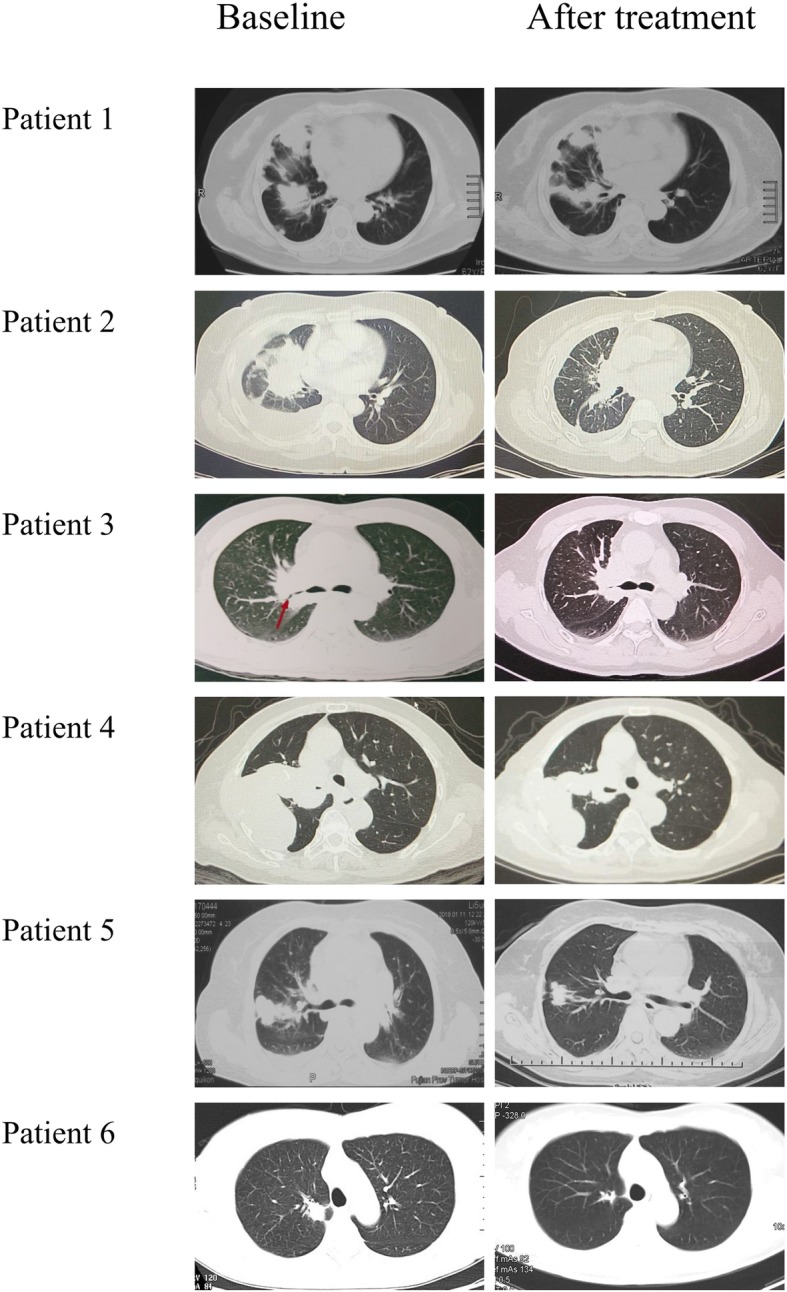
CT scans of the thorax performed before (baseline) and after osimertinib treatment (PR or SD). CT, computed tomography; PR, partial response; SD, stable disease

**Fig. 4:**
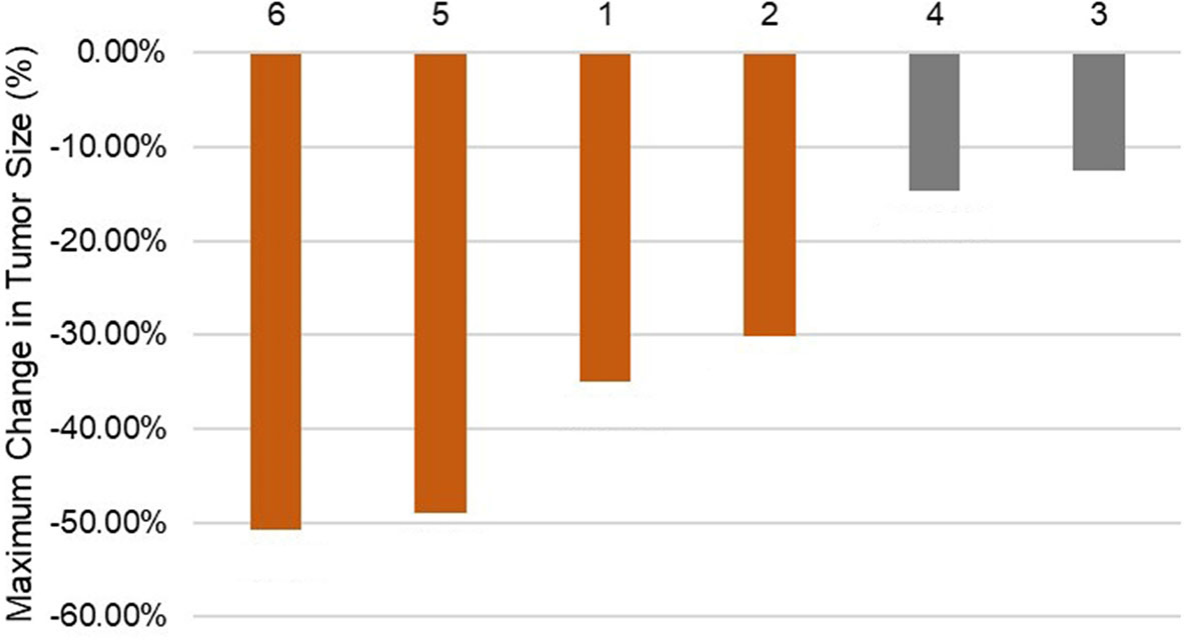
Maximum change in tumor size according to Response Criteria in Solid Tumors (RECIST) 1.1. Orange grid indicates partial response and gray grid stable disease

Patient 1 had EGFR A767_V769dup mutation and received first-line therapy with osimertinib. The patient achieved PR and the PFS was 6.0 months. PR was also observed in patient 2 (EGFR S768_D770dup), with a PFS of 14.6+ months. The patient was still on treatment at data cutoff. Patient 3 was identified with a novel EGFRex20ins mutation (EGFR N771_P772insL), which had not been reported before. The patient had SD as best response under first line osimertinib treatment and transferred to other treatment after 4.9 months due to enlarged pleural nodules. Patient 4 harbored the same EGFRex20ins mutation as that of patient 2 and attained SD with a PFS of 11.2+ months. Both patient 2 and patient 4 remained on osimertinib treatment. Patient 5 was confirmed with multiple cerebral metastasis and experienced dizzy and vomiting when diagnosed as stage IV adenocarcinoma. The patient was treated with first line chemotherapy with the best response of PD. Thereafter, the patient started osimertinib and exhibited salient clinical improvement and a reduction of nearly half the tumor burden. The patient had PD finally due to new onset bone metastasis, with a PFS of 6.4 months. Patient 6 was initially diagnosed as lung adenocarcinoma harboring EGFR A763_Y764insFQEA. The patients started gefitinib treatment with a best response of PR and PFS of 9.0 months. At disease progression, the patients had rebiopsy and his tumor was found to had EGFR A763_Y764insFQEA and EGFR T790 M. Thereafter, the patient was treated with osimertinib and attained PR. The patient experienced PD eventually due to brain metastasis and achieved a PFS of 5.1 months. CT scans performed prior to (baseline) and after osimertinib treatment are demonstrated in Fig. 3. Tumor shrinkage for each patient is shown in Fig. 4.

## Discussion

Based on a large-scale study including 2316 NSCLC patient, our study demonstrated the EGFRex20ins distribution in EGFR mutant Chinese patients (4.8%), the most common EGFRex20ins mutation (A767_V769dup) and co-mutation (TP53), as well as clinical characteristics of EGFRex20ins in Chinese NSCLC patients. As for six EGFRex20ins positive patients with osimertinib treatment, four (67.7%) patients achieved PR and two SD, with disease control rate 100%.

To our knowledge, our study represents the largest NGS based study on Chinese EGFRex20ins mutant NSCLC patients. We found that frequency of Chinese EGFRex20ins in total NSCLC (2.3%) was comparable with that of western groups in FM cohort [17]. Although the proportion of EGFR mutant patients was much larger in our Chinese cohort (47.3% vs 15.5%), which consists with previous studies [1, 2], exon 20 insertions accounted for a smaller proportion compared to FM cohort in EGFR mutant NSCLC (4.8% vs. 11.7%, *p* < 0.001) [17]. This might mainly due to different EGFR mutation proportion between Asians and Americans, rather than sequencing technology issue as discussed in a previous study [17]. In the contrast, proportion of exon 20 insertion in EGFR- mutant patients in our study is similar to those reported in other Asian cohorts, ranging from 3.6–4% [31, 32]. These results revealed a concordance in the prevalence of EGFRex20ins between Chinese and Western populations in NSCLC.

The genetic Chinese characteristics of EGFRex20ins is similar to that of western populations, including the majority of unique mutations and the most prevalent co-mutation [17]. EGFRex20ins detected in our study also tended to be exclusive with other NSCLC oncogenic drivers including ERBB2, BRAF, ALK, KRAS and RET mutations.

Most EGFRex20ins mutations (with the exception of a few subtypes such as A763_Y764insFQEA) are associated with poor responses with the 1st and 2nd generation EGFR TKIs [19–23]. Previous clinical studies including combination therapy of afatinib plus cetuximab or monotherapy of Poziotinib have demonstrated good therapeutic efficacy in some EGFRex20ins mutations positive NSCLC patients [24, 25]. However, the high proportion of severe AEs including skin toxicity and diarrhea of these therapies might limit their universal clinical applying in future. Several preclinical studies have proved that osimertinib was active in specific lung cancer cell lines with EGFRex20ins mutations [26–29], while the clinical activity of the 3rd generation EGFR TKIs in EGFRex20ins tumors remains unknown. Our study showed promising antitumor activity of osimertinib in NSCLC patients harboring certain EGFRex20ins mutations, with four patients attaining PR and two patients SD. Median PFS with osimertinib was 6.2 months, which was numerically higher than that with the 1st generation TKIs and afatinib [19, 22]. We also for the first time reported a novel EGFRex20ins mutation, EGFR N771_P772insL, in lung adenocarcinoma.

Despite the documented activity of osimertinib in our six patients, in vitro study still demonstrated limited osimertinib effect in several EGFRex20ins mutant cell lines [26–29]. Whether other EGFRex20ins mutant tumors could response to osimertinib warrants further study. These studies indicate EGFRex20ins is a heterogeneous group of EGFR mutation and deserves more researches to fully determine osimertinib sensitivity in different EGFRex20ins tumors.

A recent case report has showed that an advanced NSCLC patient with EGFRex20ins mutation, S768_D770dup, responded to osimertinib 160 mg daily [33]. The mutation detected was the same as that of patient 2 and patient 4, which might suggest favorable lower-dose osimertinib efficacy in tumors bearing EGFR S768_D770dup mutation. Another case report also suggested that EGFR H773L/V774 M, an EGFR exon 20 mutation, could be suppressed by osimertinib [34], further supporting the osimertinib effect in specific EGFR exon 20 mutations.

In addition, considering that EGFR T790 M was identified in patient 6 harboring EGFR A763_Y764insFQEA after acquired resistance to gefitinib, we found that *EGFR* T790 M served as a potential resistant mechanism in EGFR A763_Y764insFQEA positive NSCLC patients and occurrence of both mutations could be targeted by osimertinib. This is the first case showing that T790 M mediated the acquired resistance to gefitinib for patient with EGFR A763_Y764insFQEA and osimertinib treatment was effective for patient with both EGFR A763_Y764insFQEA and EGFR T790 M.

Still, there are several limitations in the study. Firstly, panels of NGS performed on patients are not uniform, contributing to relatively incomprehensive genetics statistics in patients with small panels. Secondly, although all six patients in our study acquired disease control, the sample size is too small to establish the therapeutic efficacy of osimertinib in patients with EGFRex20ins.

## Conclusions

In summary, our study revealed no significant difference in the prevalence and genetic characteristics of EGFRex20ins between Chinese and Western populations in NSCLC. Moreover, promising antitumor activity of osimertinib was observed in specific EGFRex20ins positive NSCLC patients, more studies are urgently needed to fully determine osimertinib effect in NSCLC patients with different EGFRex20ins mutations.

## Additional files

Additional file 1: **Figure S1.** EGFR ex20ins and co-mutation pattern. (JPG 186 kb)

Additional file 2: **Figure S2.** Comparison of co-mutations in EGFR ex20ins (*n* = 53), ex19del (*n* = 436), L858R (*n* = 410), T790 M (primary and secondary mutation, *n* = 58) and other EGFR sensitive mutations (*n* = 90). Others: Other EGFR sensitive mutations, including G719X, L861Q, S768I and compound mutations. (JPG 1798 kb)

## Data Availability

The datasets used and/or analyzed during the current study are available from the corresponding author on reasonable request.

## Abbreviations

AEs: Adverse events
CT: Computed tomography
EGFRex20ins: Epidermal growth factor receptor exon 20 insertion
L858R: Leu858Arg
MRI: magnetic resonance imaging
NGS: Next generation sequencing
NOS: Non-small-cell lung cancer not otherwise specified
NSCLC: Non-small-cell lung cancer
PD: Progressive disease
PFS: Progression-free survival
PR: Partial response
RECIST: Response criteria in solid tumors
SD: Stable disease
TKIs: Tyrosine kinase inhibitors
