# Supplementary figures and images for: EGFR exon 20 insertion mutations and response to osimertinib in non-small-cell lung cancer
Source: BMC Cancer. 2019 Jun 17;19:595. doi: 10.1186/s12885-019-5820-0 (PMC6580637; doi:10.1186/s12885-019-5820-0)

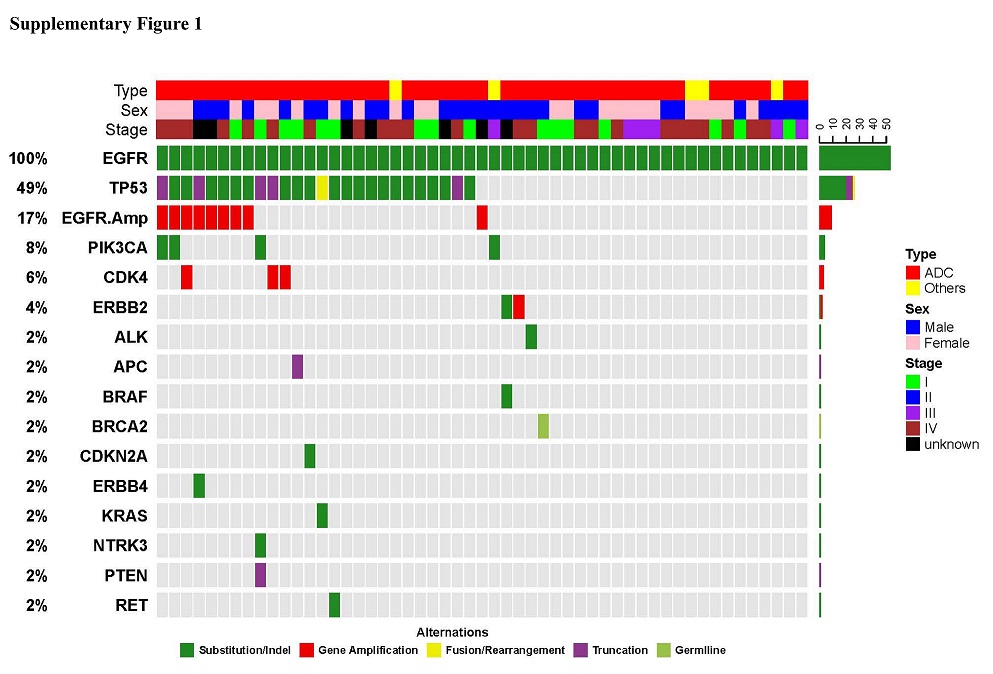

Supplement: Supplementary file 1 — Figure S1. EGFR ex20ins and co-mutation pattern. (JPG 186 kb) [file 12885_2019_5820_MOESM1_ESM.jpg]

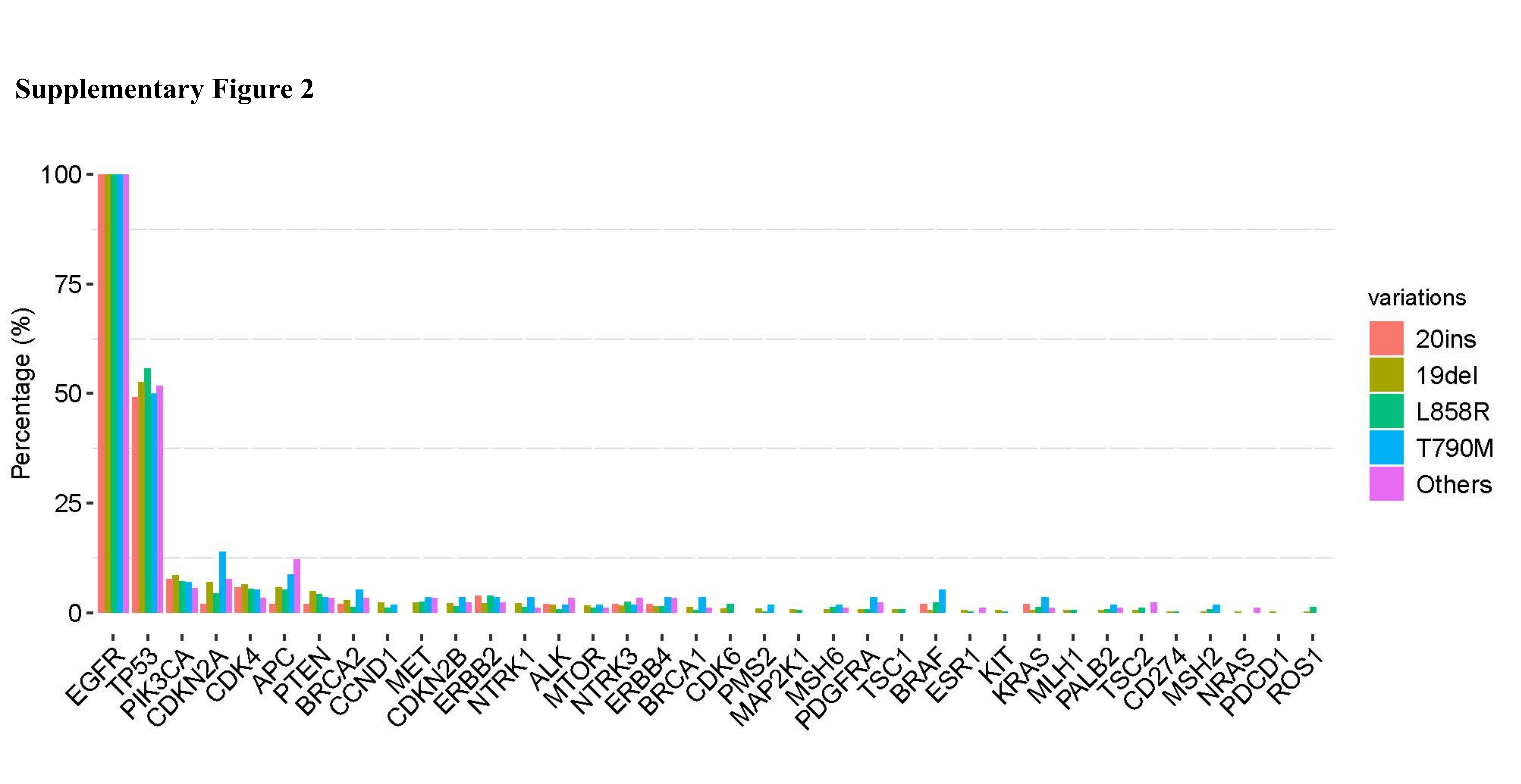

Supplement: Supplementary file 2 — Figure S2. Comparison of co-mutations in EGFR ex20ins (n = 53), ex19del (n = 436), L858R (n = 410), T790 M (primary and secondary mutation, n = 58) and other EGFR sensitive mutations (n = 90). Others: Other EGFR sensitive mutations, including G719X, L861Q, S768I and compound mutations. (JPG 1798 kb) [file 12885_2019_5820_MOESM2_ESM.jpg]
